# Supplementary material for: Factors predicting the visual outcome of intracorneal ring segment for keratoconus
Source: PLoS One. 2024 Feb 6;19(2):e0288181. doi: 10.1371/journal.pone.0288181 (PMC10846708; doi:10.1371/journal.pone.0288181)
Supplement: S1 Checklist — (DOCX) [file pone.0288181.s001.docx]

STROBE Statement—checklist of items that should be included in reports of observational studies

|  | Item No. | Recommendation | Page  No. | Relevant text from manuscript |
| --- | --- | --- | --- | --- |
| **Title and abstract** | 1 | (*a*) Indicate the study’s design with a commonly used term in the title or the abstract | 2 | Medical records of 287 keratoconic eyes implanted with ICRS at Chula Refractive Surgery Centre of a tertiary university hospital (Bangkok, Thailand) between January 2012 and March 2022 were **retrospectively** reviewed for epidemiological and clinical preoperative variables, including those derived from Scheimpflug tomography. |
|  |  | (*b*) Provide in the abstract an informative and balanced summary of what was done and what was found | 2 |  |
| Introduction | | | |  |
| Background/rationale | 2 | Explain the scientific background and rationale for the investigation being reported | 4 |  |
| Objectives | 3 | State specific objectives, including any prespecified hypotheses | 5 | we aimed to identify predictors of visual outcomes at 6 months (or within 5 – 12 months) following ICRS implantation. We also created a mathematical model using the abovementioned factors to predict postoperative visual acuities quantitatively. |
| Methods | | | |  |
| Study design | 4 | Present key elements of study design early in the paper | 5 | This retrospective study was conducted during November 2022 |
| Setting | 5 | Describe the setting, locations, and relevant dates, including periods of recruitment, exposure, follow-up, and data collection | 5-6 | by collecting data from medical records of patients who underwent ICRS implantation at the Chula Refractive Surgery Center, King Chulalongkorn Memorial Hospital, Bangkok, Thailand. The primary objective was to identify predictors of postoperative visual acuity change 6 months (or within 5-12 months) following ICRS implantation in patients with keratoconus. |
| Participants | 6 | (*a*) *Cohort study*—Give the eligibility criteria, and the sources and methods of selection of participants. Describe methods of follow-up  *Case-control study*—Give the eligibility criteria, and the sources and methods of case ascertainment and control selection. Give the rationale for the choice of cases and controls  *Cross-sectional study*—Give the eligibility criteria, and the sources and methods of selection of participants | 5-6 | The medical records of all eligible cases (351 eyes) were included in this study. The inclusion criteria were as follows: patients with keratoconus implanted with ICRS (Ferrara Ring, AJL, Boecillo, Spain) at the Chula Refractive Surgery Centre between January 2012 and March 2022. … The exclusion criteria were as follows: (i) lack of visual acuity measurement during 5 - 12 months after ICRS implantation (39 eyes), (ii) absence of preoperative data (14 eyes), and (iii) vision-affecting complication and/or ICRS removal within the follow-up period (11 eyes). |
|  |  | (*b*) *Cohort study*—For matched studies, give matching criteria and number of exposed and unexposed  *Case-control study*—For matched studies, give matching criteria and the number of controls per case |  |  |
| Variables | 7 | Clearly define all outcomes, exposures, predictors, potential confounders, and effect modifiers. Give diagnostic criteria, if applicable | 6-7 | The epidemiological, pre-implantation, and post-implantation data were obtained from patients’ medical records. … The post-implantation visual outcome was assessed by determining changes in the UDVA and CDVA at six months (or within 5-12 months) post-surgery and comparing them to preoperative values (ΔUDVA and ΔCDVA). |
| Data sources/ measurement | 8* | For each variable of interest, give sources of data and details of methods of assessment (measurement). Describe comparability of assessment methods if there is more than one group | 6-7 |  |
| Bias | 9 | Describe any efforts to address potential sources of bias | - |  |
| Study size | 10 | Explain how the study size was arrived at | - |  |

Continued on next page

| Quantitative variables | 11 | Explain how quantitative variables were handled in the analyses. If applicable, describe which 6-8groupings were chosen and why | 6-8 |  | |
| --- | --- | --- | --- | --- | --- |
| Statistical methods | 12 | (*a*) Describe all statistical methods, including those used to control for confounding | 7-8 |  | |
|  |  | (*b*) Describe any methods used to examine subgroups and interactions | 7-8 |  | |
|  |  | (*c*) Explain how missing data were addressed | 6 | The exclusion criteria were as follows: (i) lack of visual acuity measurement during 5 - 12 months after ICRS implantation (39 eyes), (ii) absence of preoperative data (14 eyes), | |
|  |  | (*d*) *Cohort study*—If applicable, explain how loss to follow-up was addressed  *Case-control study*—If applicable, explain how matching of cases and controls was addressed  *Cross-sectional study*—If applicable, describe analytical methods taking account of sampling strategy | 7-8 |  | |
|  |  | (*e*) Describe any sensitivity analyses |  |  | |
| Results | | | | |  |
| Participants | 13* | (a) Report numbers of individuals at each stage of study—eg numbers potentially eligible, examined for eligibility, confirmed eligible, included in the study, completing follow-up, and analysed | 5-6 | The medical records of all eligible cases (351 eyes) were included in this study. … Ultimately, 64 eyes were excluded from the study, yielding a study cohort of 287 eyes. | |
|  |  | (b) Give reasons for non-participation at each stage | 6 | The exclusion criteria were as follows: (i) lack of visual acuity measurement during 5 - 12 months after ICRS implantation (39 eyes), (ii) absence of preoperative data (14 eyes), and (iii) vision-affecting complication and/or ICRS removal within the follow-up period (11 eyes). | |
|  |  | (c) Consider use of a flow diagram | - |  | |
| Descriptive data | 14* | (a) Give characteristics of study participants (eg demographic, clinical, social) and information on exposures and potential confounders | 8 | There were 161 males (70.0%) and 69 females (30.0%). The mean age at onset was 20.11 ± 7.86 years (range: 7–50 years), while the mean age at ring implantation time was 26.97 ± 8.56 years (range: 13–59 years). | |
|  |  | (b) Indicate number of participants with missing data for each variable of interest | 10 | (Table1) | |
|  |  | (c) *Cohort study*—Summarise follow-up time (eg, average and total amount) |  |  | |
| Outcome data | 15* | *Cohort study*—Report numbers of outcome events or summary measures over time | *-* |  | |
|  |  | *Case-control study—*Report numbers in each exposure category, or summary measures of exposure | - |  | |
|  |  | *Cross-sectional study—*Report numbers of outcome events or summary measures | 8-11 |  | |
| Main results | 16 | (*a*) Give unadjusted estimates and, if applicable, confounder-adjusted estimates and their precision (eg, 95% confidence interval). Make clear which confounders were adjusted for and why they were included | 14-15 | (Table 4)(Table 5) | |
|  |  | (*b*) Report category boundaries when continuous variables were categorized | - |  | |
|  |  | (*c*) If relevant, consider translating estimates of relative risk into absolute risk for a meaningful time period | - |  | |

Continued on next page

| Other analyses | 17 | Report other analyses done—eg analyses of subgroups and interactions, and sensitivity analyses | - |  |
| --- | --- | --- | --- | --- |
| Discussion | | | | |
| Key results | 18 | Summarise key results with reference to study objectives | 16-20 | According to our literature review, this may be the largest retrospective study of the relationship between ICRS-induced visual acuity changes and various demographic and preoperative factors of patients with keratoconus. The diversity of studied variable types was another strength of our study. This study was also the first attempt to create an equation to predict ΔUDVA following ICRS implantation. In the multiple linear regression analysis, UDVAp and PIavg seemed to be good predictors for ΔUDVA. |
| Limitations | 19 | Discuss limitations of the study, taking into account sources of potential bias or imprecision. Discuss both direction and magnitude of any potential bias | 19-20 |  |
| Interpretation | 20 | Give a cautious overall interpretation of results considering objectives, limitations, multiplicity of analyses, results from similar studies, and other relevant evidence | 16-20 |  |
| Generalisability | 21 | Discuss the generalisability (external validity) of the study results | 19-20 | The utility of the proposed models might be limited because its variables contained the spherical power from autorefraction, which is frequently unmeasurable in advanced keratoconus. |
| Other information | |  | | |
| Funding | 22 | Give the source of funding and the role of the funders for the present study and, if applicable, for the original study on which the present article is based | - |  |

*Give information separately for cases and controls in case-control studies and, if applicable, for exposed and unexposed groups in cohort and cross-sectional studies.

**Note:** An Explanation and Elaboration article discusses each checklist item and gives methodological background and published examples of transparent reporting. The STROBE checklist is best used in conjunction with this article (freely available on the Web sites of PLoS Medicine at http://www.plosmedicine.org/, Annals of Internal Medicine at http://www.annals.org/, and Epidemiology at http://www.epidem.com/). Information on the STROBE Initiative is available at www.strobe-statement.org.
