# Supplementary material for: Factors predicting the visual outcome of intracorneal ring segment for keratoconus
Source: PLoS One. 2024 Feb 6;19(2):e0288181. doi: 10.1371/journal.pone.0288181 (PMC10846708; doi:10.1371/journal.pone.0288181)
Supplement: S1 Table — (DOCX) [file pone.0288181.s002.docx]

**Supplementary table 1. Intraclass Correlation (ICC) assessment to quantify the degree of similarity between the measurements from the two eyes in the bilateral cases (n = 57).**

| Variables | Single measured ICC | No. of targets | p | Variables | Single measured ICC | No. of targets | p |
| --- | --- | --- | --- | --- | --- | --- | --- |
| IOP | 0.622 | 35 | 0.000 | DAT | 0.639 | 57 | 0.000 |
| UDVAp | 0.339 | 57 | 0.004 | DAP | 0.673 | 57 | 0.000 |
| CDVAp | 0.191 | 57 | 0.075 | DAK | 0.750 | 57 | 0.000 |
| SPH | 0.731 | 42 | 0.000 | DTK | 0.403 | 57 | 0.001 |
| CYL | 0.299 | 42 | 0.025 | DTP | 0.761 | 57 | 0.000 |
| Kmfront | 0.219 | 57 | 0.048 | DPK | 0.774 | 57 | 0.000 |
| Kmback | 0.349 | 57 | 0.004 | ISV | 0.508 | 57 | 0.000 |
| Kmax | 0.427 | 57 | 0.000 | IVA | 0.699 | 57 | 0.000 |
| KF | 0.634 | 57 | 0.000 | KI | 0.698 | 57 | 0.000 |
| CA | 0.714 | 57 | 0.000 | CKI | 0.557 | 57 | 0.000 |
| Qfront | 0.149 | 57 | 0.130 | IHA | 0.468 | 57 | 0.000 |
| Qback | 0.594 | 57 | 0.000 | IHD | 0.629 | 57 | 0.000 |
| CPT | 0.776 | 57 | 0.000 | Rmin | 0.507 | 57 | 0.000 |
| CAT | 0.656 | 57 | 0.000 | IS | 0.790 | 57 | 0.000 |
| CTT | 0.627 | 57 | 0.000 | RMStotal | 0.542 | 57 | 0.000 |
| BADD | 0.360 | 57 | 0.000 | LOA | 0.537 | 57 | 0.000 |
| PIavg | 0.328 | 57 | 0.006 | HOA | 0.568 | 57 | 0.000 |
| ARTmax | 0.317 | 57 | 0.007 |  |  |  |  |

**Abbreviations:** IOP, intraocular pressure; UDVAp, preoperative uncorrected distance visual acuity; CDVAp, preoperative corrected distance visual acuity; SPH, spherical power; CYL, cylindrical power; Km-front, mean keratometry of front surface; Km-back, mean keratometry of back surface; KF, K factor; Kmax, maximum keratometry; CA, anterior corneal astigmatism; Q-front, Q-value of front surface; Q-back, Q-value of back surface; CPT, thickness at the pupil center; CAT, thickness at the apex; CTT, thickness at the thinnest point; BAD D, Belin/Ambrósio enhanced ectasia display; PIavg, average pachymetric progression index; ARTmax, Ambrósio relational thickness maximum; DAT: distance from apex to thinnest point; DAP: distance from apex to pupil center; DAK: distance from apex to maximum keratometry point; DTK: distance from thinnest point to maximum keratometry point; DTP: distance from thinnest point to pupil center; DPK: distance from pupil center to maximum keratometry point; ISV, index of surface variance; IVA, index of vertical asymmetry; KI, keratoconus index; CKI, central keratoconus index; IHA, index of height asymmetry; IHD, index of height decentration; Rmin, anterior minimum sagittal curvature; rms, root mean square; LOA, corneal lower order aberration; HOA, corneal higher order aberration.
