# Supplementary material for: Factors predicting the visual outcome of intracorneal ring segment for keratoconus
Source: PLoS One. 2024 Feb 6;19(2):e0288181. doi: 10.1371/journal.pone.0288181 (PMC10846708; doi:10.1371/journal.pone.0288181)
Supplement: Attachment — Submitted filename: Response to reviewers.docx [file pone.0288181.s004.docx]

**Review Comments to the Author**

Reviewer #1: This is an interesting and well - written manuscript.

The primary concern here is the violation of the assumption of independence of observations, a principle that is fundamental to many statistical tests. By including both eyes from the same patient, the independence of data points is jeopardised, potentially leading to an increased risk of Type I errors.

I would like to recommend a few potential solutions to address this issue. You may consider:

1) Including data from only one eye per patient (I recommend this option - 230 eyes from 230 patients).

2) If data from both eyes are essential to your analysis, more complex statistical approaches such as Generalised Estimating Equations (GEE) or Mixed Effects Models can account for the correlation between the two eyes.

3) Performing an Intraclass Correlation (ICC) assessment to quantify the degree of similarity between the measurements from the two eyes.

Given these methodological concerns, I strongly recommend seeking the advice of a professional biostatistician. Their expertise will be crucial in ensuring that your analysis appropriately addresses these issues and strengthens the credibility of your findings.

**Response:** Thank you for pointing this out. To enhance the credibility of our findings according to these comments, we have consulted a professional biostatistician following your recommendation. The intraclass correlation (ICC) was explored, and we found that there is high similarity between the measurements from the two eyes. Therefore, we decide to recruit only one eye per patient to eliminate the dependency of observations, so the more complex statistical approaches such as Generalised Estimating Equations (GEE) or Mixed Effects Models are not further mandatory. The results of regression analysis have changed as the newly obtained candidate variables altered.

Reviewer #2: The manuscript by Khanthik et al. performs a retrospective analysis of KC patients that received ICRS implants to determine a predictive model for a patient to receive ICRS implantation. Their analysis included various important components including biological factors and various factors to determine diagnose KC including spherical power, astigmatism, keatometry of front and back surface, and thinnest region of eye. Additionally, their discussion included various studies that both agreed and disagreed with their finds. Finally, they included the limitations of their studies including the short term aspect and limited potential in severe KC cases.

**Response:** Thank you
